# EGR-2 Is Not Required for In Vivo CD4 T Cell Mediated Immune Responses

**DOI:** 10.1371/journal.pone.0012904

**Published:** 2010-09-23

**Authors:** Hilda E. Ramón, Pedro J. Cejas, David LaRosa, Adeeb Rahman, John E. Harris, Jidong Zhang, Christopher Hunter, Yongwon Choi, Laurence A. Turka

**Affiliations:** 1 Department of Medicine, University of Pennsylvania School of Medicine, Philadelphia, Pennsylvania, United States of America; 2 Department of Pathology and Laboratory Medicine, University of Pennsylvania School of Medicine, Philadelphia, Pennsylvania, United States of America; 3 Department of Dermatology, University of Pennsylvania School of Medicine, Philadelphia, Pennsylvania, United States of America; 4 Department of Pathobiology, University of Pennsylvania School of Veterinary Medicine, Philadelphia, Pennsylvania, United States of America; Oklahoma Medical Research Foundation, United States of America

## Abstract

**Background:**

The zinc finger transcription factor EGR-2 has been shown to play an important role in the induction of T cell anergy and the regulation of peripheral T cell tolerance. *In vitro*, a prior study has show that T cells deficient in EGR-2 are hyperproliferative to IL-2 and produce elevated levels of the effector cytokine IFN-γ. EGR-2 deficient mice have increased levels of CD44^high^ T cells in peripheral lymphoid organs, and with age, develop autoimmune-like features.

**Principal Findings:**

Here we show that despite increased numbers of cells bearing an activated CD44^high^CD62L^low^ phenotype, T cells from young healthy EGR-2 deficient mice have normal proliferative and cytokine responses, and the mice themselves mount normal immune responses against minor histocompatibility antigens, and the pathogens *Toxoplasma gondii* and lymphocytic choriomeningitis virus.

**Conclusions:**

Our results indicate that EGR-2 is not required to mount normal acute *in vivo* immune responses against foreign antigens, and suggest instead that it may serve to regulate the response to chronic antigenic exposure, such as that which occurs to autoantigens.

## Introduction

The early growth response (EGR) family of zinc finger transcription factors includes 4 known members, which have important functions in the central nervous system, cancer, hematopoiesis, and immunological tolerance [1]–[4]. These transcription factors are expressed in T cells; among these, EGR1–3 are upregulated after T cell receptor (TCR) stimulation [5], [6].

While EGR-1 has been shown to be a positive regulator of T cell activation [7], [8], EGR-2 and EGR-3 have been shown to play a role in T cell anergy induction [4], [9]–[11]. *In vitro*, both EGR-2 and EGR-3 are highly expressed in T cells activated under anergizing conditions, such as stimulation through the T cell receptor in the absence of costimulation or exposure to ionomycin [10]–[11], and their expression is blocked by the calcineurin inhibitor cyclosporine A, which prevents anergy induction in T cells [6]. EGR-2 mRNA and protein levels, unlike those of EGR-3, remain upregulated for days following anergy induction, suggesting that it has a significant role in the induction of T cell anergy [10]–[11]. Moreover, treatment of T cells with siRNA to inhibit EGR-2 expression restores IL-2 production and proliferation of in vitro anergized T cells, indicating that EGR-2 is required for anergy induction [10].

EGR-2 has also been implied to play an important role in T cell tolerance *in vivo*. T cells specific for the influenza hemagglutinin antigen (HA) transferred into the tolerizing model of C3-HA mice, which express HA as a self-antigen, are induced to express high levels of EGR-2 [11]. More importantly, mice with T cell specific EGR-2 deficiency have increased numbers of CD44^high^ T cells, and develop an autoimmune-like illness with anti-DNA and anti-histone antibodies, elevated serum IgG2a, glomerulonephritis, inflammatory infiltrates in the liver and kidney, and skin lesions, and with its onset at 1 year of age and a median survival of 15 months. This syndrome was shown to be T cell-driven, as demonstrated by the transfer of EGR-2 deficient T cells into RAG2−/− mice [12].

Although EGR-2 does not seem to play a role in the initial steps of T cell activation, EGR-2 deficient T cells were observed to be hyperresponsive to exogenous IL-2 following primary stimulation [12] and to express increased amounts of the effector cytokines IFN-γ and IL-17 after activation [12]. Furthermore, EGR-2 has been shown to induce the expression of FasL in activated T cells [13], [14]. Given these results it has been proposed that EGR-2 might also regulate effector T cell responses, in addition to tolerance induction [4], [12].

The role of EGR-2 during a T cell response *in vivo* has not been previous reported. Here, using mice with T cell specific targeting of EGR-2, we have tested its T cell intrinsic role during *in vivo* T cell responses including those to minor histocompatibility antigens, and LCMV and *Toxoplasma gondii* infections. Contrary to our hypothesis that EGR-2 deficiency would lead to exaggerated T cells responses, we find that EGR-2 deficient mice mount a normal *in vivo* T cell responses with a peak and contraction face comparable to that seen in wild-type animals.

## Results

### Increased levels of CD44 on T cells from EGR-2 conditional knockout mice

**Figure 1:**
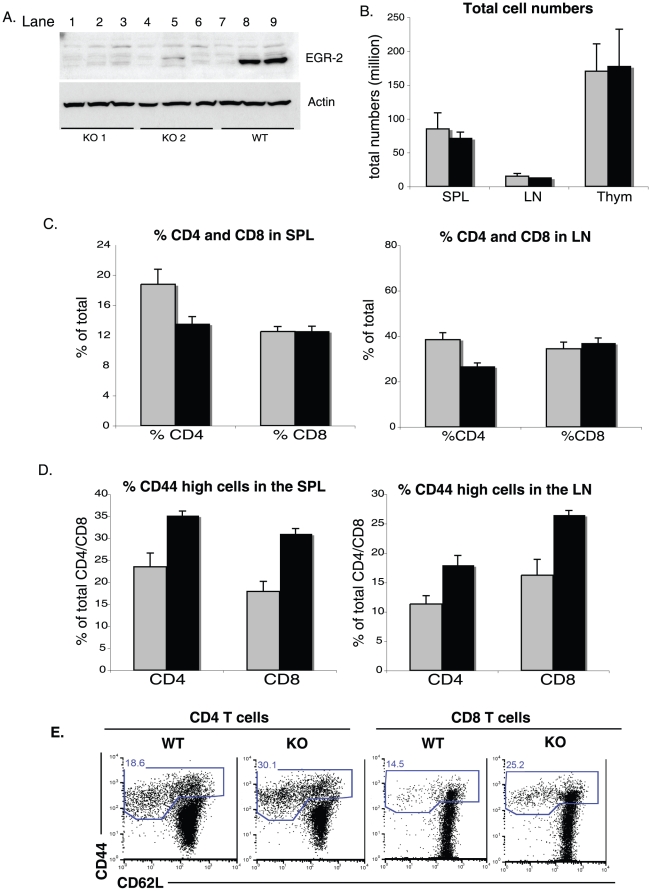
EGR-2 deficient mice have elevated levels of CD44 high T cells. (A) Western blot analysis of lysates from MACS-purified CD4 T cells that left untreated (lanes 1,4,7) or stimulated for 15 hours with CD3 (1µg/mL) (lanes 2,5,8) or CD3 (1µg/mL) and CD28 (5µg/mL) (lanes 3,6,9). (B) Total cell counts from WT (gray) or EGR-2 deficient mice (black). (C) Percentages of CD4^+^ and CD8^+^ T cells in spleen (SPL) and lymph nodes (LN), which were obtained through flow cytometric analysis. (D) Percentages of CD44^high^ cells from spleen and lymph nodes analyzed through flow cytometry and calculated after gating on CD4^+^ or CD8^+^ T cells. All graphs show results from three mice from each genotype. (E) Levels of CD44 and CD62L for CD4 and CD8 T cells in spleen of EGR-2 CKO or WT mice. Gates represent the percentage of activated cells.

As previously reported, EGR-2 deficient mice are perinatally lethal [15], therefore, in order to analyze the role of EGR-2 in T cell responses *in vivo*, we crossed EGR-2^flox/flox^ mice to CD4-*Cre* mice (mice used for subsequent studies reported in this paper were backcrossed onto a B6 background for at least 10 generations). Western blot analysis of the *Cre*-expressing progeny confirmed the absence of detectable EGR-2 protein in activated T cells (Figure 1a). Consistent with a prior report [12], analysis of the spleen, lymph node and thymus showed similar cellularity in EGR-2 deficient mice compared to WT controls, suggesting no obvious defect in lymphocyte development (Figure 1b). Further analysis of the spleen and lymph nodes shows similar percentages of CD8 T cells and a small decrease in the percentage of CD4 T cells in both of these organs (Figure 1c). We noted that T cell specific Egr-2 deficient mice have higher percentages of CD4^+^CD44^high^ and CD8^+^CD44^high^ T cells in spleen and lymph nodes (Figure 1d and 1e), indicative of the accumulation of activated cells in secondary lymphoid tissue. This finding is consistent with a previous study [12], although occurred at a somewhat earlier age in our colony, and suggests suggests that EGR-2 may play a role in the regulation of T cell activation or effector T cell homeostasis.

### EGR-2 deficient T cells show normal proliferation and cytokine production

**Figure 2:**
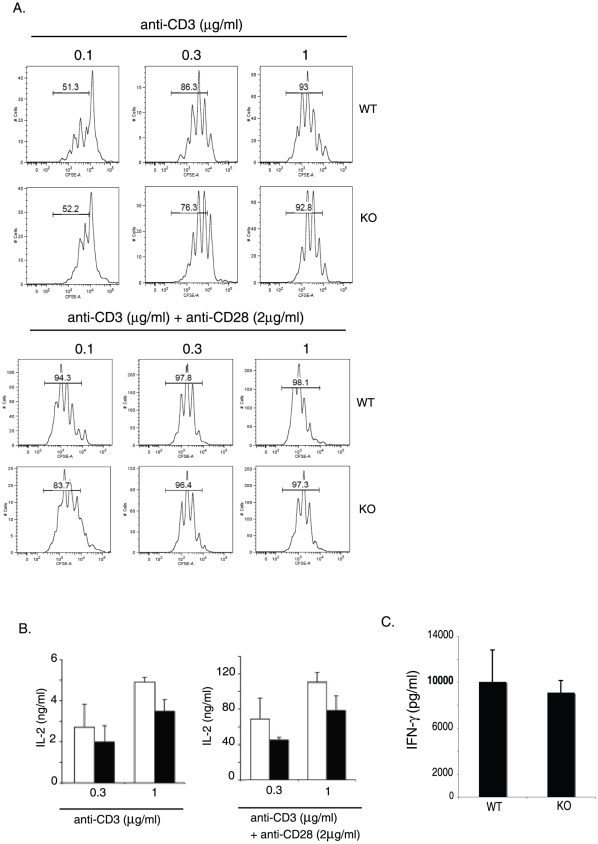
EGR-2 deficient T cells show normal proliferation and cytokine production. (A) CFSE analysis of purified CD4 T cells after activation with anti-CD3 alone or anti-CD3 and anti-CD28 at the specified conditions. (B) IL-2 production from the cultures described above, measured through ELISA. (C) IFN-γ production of purified CD4 T cells after activation with anti-CD3 and antigen presenting cells, measured by ELISA.

The observation of increased cytokine production of EGR-2-deficient T cells [12], along with the presence of elevated numbers of CD44^high^ cells in EGR-2-deficient mice (ref. 12 and Figure 1), led us to hypothesize that EGR-2 CKO mice would have exaggerated T cell responses. To test this, we first analyzed the response of purified T cells from 3 month old mice following *in vitro* stimulation with plate-bound antibodies against CD3 +/− CD28. Despite the elevation in the percentage of activated T cells (Figure 1), and consistent with previously published data [12], EGR-2 deficient CD4 T cells showed normal proliferation and IL-2 production in response to TCR stimulation (Figure 2a and 2b). IFN-γ production was also similar between EGR-2 KO and WT CD4 T cells, after activation with anti-CD3 plus APCs antigen presenting cells (APC) for 3 days (Figure 2c).

### T cell response to minor histocompatibility antigens

**Figure 3:**
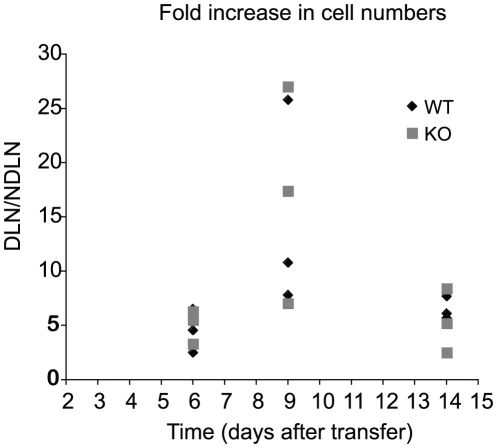
EGR-2 deficient female mice mount a normal response against the Y antigens. Female WT or EGR-2 deficient mice were injected in the footpad with 20 million total splenocytes from WT male mice. The number of cells in the draining (DLN) and non-draining (NDLN) lymph nodes was calculated and the ratio of DNL/DNLN is shown as fold increase in cellularity. Each condition includes three mice of each genotype.

To begin to analyze *in vivo* responses in EGR-2 CKO mice, we first used examined the response to allogeneic antigens using a minor histocompatibilty mismatch model. Proteins expressed on the Y chromosome are only found in males and can be recognize by the immune system of female mice as alloantigens, inducing an immune response that is mediated by both CD4 and CD8 T cells. We injected cells from male mice into the footpad of female mice and followed the immune response by analyzing the increase in cell numbers in the draining lymph node, compared to the non-draining lymph node (Figure 3). At day six after the footpad injection we observed a roughly six fold increase in cells in the draining lymph node (compared to cell numbers in the contralateral non-draining node). This response was seen to peak at day 9, with up to a 28 fold increase in cell numbers. A comparable increase in cell numbers was seen in both the WT and CKO mice, indicating that EGR-2 deficient mice are capable of mounting a normal immune response against minor histocompatibility antigens. Surprisingly, examination of cell numbers at day 14 revealed a similar physiologic contraction of cell numbers, demonstrating normal resolution of the immune response in EGR-2-deficient T cells.

### T cell response to *Toxoplasma gondii*

**Figure 4:**
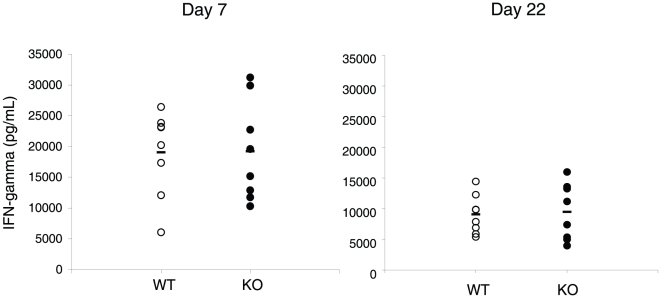
EGR-2 deficient mice produce normal levels of INF-g in response to *T. gondii* infection. WT (white) or EGR-2 CKO (black) mice were infected with *T. gondii*. The levels of IFN-γ in the serum were measured on days 7 and 22 through ELISA. Each circle represents a single mouse.

*Toxoplasma gondii* is a parasite that induces strong T_H_-1 type responses mediated by both CD4 and CD8 T cells [16], which produce high levels of IFN-γ [17]. In order to test the response to this parasite in EGR-2 deficient mice, 8 CKO and 8 WT mice were infected with *Toxoplasma gondii* intraperitoneally and serum IFN-γ levels were measured at days 7 and 22 after infection (Figure 4). As expected, infection with *T. gondii* induced high levels of serum IFN-γ in control mice at day 7 after infection. EGR-2 deficient mice showed similar levels of this cytokine, indicating that they mounted a normal response against this parasite. Furthermore, the levels of IFN-γ were similarly decreased by day 22 in both WT and CKO mice, also indicating that EGR-2 deficient mice had normal resolution of the response.

### T cell response to LCMV

**Figure 5:**
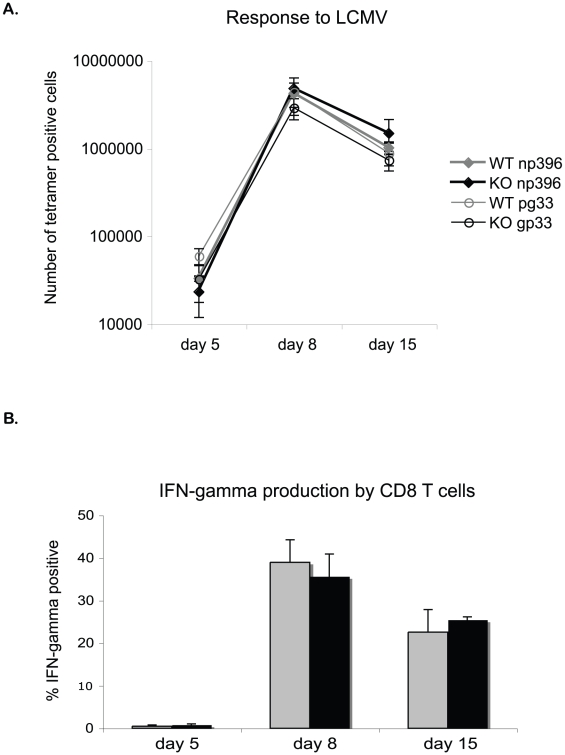
EGR-2 deficient mice mount a normal T cell response against LCMV. WT or EGR-2 CKO mice were infected with 2×10^5^ PFU of LCMV Armstrong. (A) The number of CD8 T cells specific for gp33 or np396 was measured by staining with MHC-class I tetramers, on days 5, 8, and 15. (B) The percentage of CD8 cells that were IFN-γ producers was analyzed by intracellular cytokine staining after restimulation with LCMV pooled peptide. For all graphs, three mice were analyzed for each genotype on each time point.

We next tested the response of EGR-2-deficient T cells to LCMV. LCMV is a natural mouse pathogen that elicits a strong virus-specific CD8 T cell response characterized by high levels of IFN-γ [18]. This is a particularly useful model system as it enables the enumeration of antigen specific T cell populations using MHC-class I tetramers. WT or EGR-2 CKO mice were infected with the Armstrong strain of LCMV, which induces an acute infection, and the virus-specific response was followed using tetramers to identify CD8 T cells specific for the gp33 and np396 immunodominant epitopes. On day 8 of infection the number of gp33 and np396-specific CD8 T cells in the spleen had increased in both WT and EGR-2 CKO mice, compared to that on day 5 (Figure 5a). As the numbers of tetramer positive cells on day 5 were no different than that of an un-infected control (data not shown), this indicates that there was a normal expansion of virus-specific CD8 T cells in EGR-2 CKO mice. Moreover, the numbers of gp33 and np396-specific CD8 T cells showed a similar decline by day 15 in both WT and EGR-2 CKO mice, indicating a normal contraction phase.

In addition to enumerating antigen responsive T cells we also tested their function as assessed by IFN-γ production following *ex vivo* restimulation with LCMV pooled peptide. Eight days after infection, at the peak of the response, there was significant induction (compared with day 5) of IFN-γ production by CD8 T cells in both WT and EGR-2 CKO mice, which correlates with an increase in the number of LCMV-specific CD8 T cells (Figure 5b). By day 15 after infection, the percentages of IFN-γ positive cells had declined in both sets of mice, reflecting the drop in tetramer positive CD8 T cell frequency. These data further indicate that the expansion, peak and contraction phase during a response to LCMV are normal in EGR-2 deficient mice.

## Discussion

EGR-2 has been implicated in the induction CD4 T cell anergy, and the regulation of peripheral tolerance *in vivo*. A previous study has reported that mice that are deficient in EGR-2 show symptoms of autoimmunity and have elevated levels of activated CD4 T cells in peripheral lymphoid organs [12], suggesting that this transcription factor plays an important role in the prevention of T cell responses against self antigens. Those investigators also found that CD4 T cells from EGR-2 deficient mice were hyperproliferative in response to IL-2 re-stimulation and expressed higher levels of effector cytokines such as IFN-γ and IL-17 *in vitro* [12], which suggested that EGR-2 might also play a role in the regulation of CD4 T cells responses *in vivo*. However, the results we present in this report indicate that EGR-2 deficient mice mount a normal immune T cell immune response *in vivo*, comparable to that of WT mice.

Using a model of minor histocompatibility mismatch, we found that EGR-2 deficient mice show a normal increase in cellularity in draining lymph nodes and a normal contraction phase, as compared to WT littermates. We have also shown that in response to *T. gondii* infection, EGR-2 CKO mice have a physiologic increase in IFN-γ levels in the serum at the peak of the response, and these decrease normally during the contraction phase. Lastly, using tetramers to analyze antigen-specific CD8 T cells we observed that after LCMV infection, EGR-2 deficient mice have a normal increase in CD8 T cells specific to two major epitopes, gp33 and np396. The number of antigen-specific CD8 T cells also declined appropriately during the contraction phase. Furthermore, the percentages of IFN-γ secreting cells in response to LCMV were comparable between EGR-2 CKO mice and WT littermates at different stages of infection.

These results differ in some respects from those of Zhu et al. [12], in that we did not observe an increase in IFN-γ production in EGR-2 deficient CD4 T cells. While the reasons for this are not immediately apparent, they could be due to differing stimulation conditions and assays, or the fact that the mice used in our studies were more extensively backcrossed onto a B6 background, which tends to be less autoimmune prone.

Our results indicate that EGR-2 deficient mice can mount normal *in vivo* T cell responses when they are challenged with three different classes of antigens – minor histocompatibility antigen, parasite and viral antigens. All of these are non-self antigens, and these findings contrast with published evidence indicating a role for EGR-2 in regulating tolerance to self-antigens [12]. It is tempting to speculate that the difference in the role of EGR-2 in the response to foreign vs. self-antigens may lie in the different contexts (inflammatory vs. non-inflammatory) in which foreign and self-antigens are typically presented. However it is important to note that all of our studies were performed on young healthy mice. It is certainly possible that older mice with autoimmunity would have dysregulated responses to foreign antigens. Thus our primary conclusion is that loss of EGR-2 in T cells does not, per se, interfere with a normal response to pathogens in otherwise healthy animals.

## Materials and Methods

### Mice

EGR-2 floxed mice have been previously described [19]. They have been crossed to the B6 background for more than 10 generations. CD4 Cre mice were purchased from The Jackson Laboratories. Mice were kept in a barrier facility at the University of Pennsylvania, and transferred to a BSL2 facility prior to the T. gondii and LCMV infections. In all experiments, mice used were 2–3 months of age, prior to the onset of autoimmune symptoms.

### Western Blot

MACS-purified CD4 T cells from spleen and lymph nodes of either WT or EGR-2 CKO mice were cultured for 15 hours in complete media alone or with plate-bound anti-CD3 (1µg/mL) or both anti-CD3 (1µg/mL) and anti-CD28 (5µg/mL). Cell lysates were analyzed as described in [20] for the presence of EGR-2 protein using an anti-EGR-2 antibody (Covance), or anti-actin as control.

### CD4 T cell culture and measurement of cytokine production

CD4 T cells were MACS purified using a negative selection kit from Miltenyi. T cells were CFSE labeled and culture at the stated concentrations of anti-CD3 +/− anti-CD28. Proliferation was analyzed after three days of culture. IL-2 was analyzed from supernatants obtained after 20 hours of culture and measured through ELISA. IFN-γ was analyzed from supernatants taken after three days of culture and analyzed by ELISA.

### Minor histocompatibility mismatch responses

Female WT or EGR-2 CKO mice were injected in the right footpad with 20 million splenocytes from male WT mice in 50µl of PBS. On days 6, 9 and 14 after injection the number of cells in the draining (right) versus non-draining (left) lymph nodes were calculated.

### *Toxoplasma gondii* infection

WT and EGR-2 CKO mice were injected intraperitoneally with *Toxoplasma gondii*, using 20 cysts per mouse in 100µl of PBS. Mice were bled at days 7 and 22 after infection. Levels of IFN-γ in the serum were measured through ELISA (BD Biosciences Kit).

### LCMV infection

WT and EGR-2 CKO mice were injected intraperitoneally with the Armstrong strain of LCMV (gift from Dr. John Wherry). Each mouse received 2×10^5^ PFU in 500µl of PBS. On days 5, 8 and 15, spleens were collected. The number of tetramer positive cells in the spleen was analyzed using allophycocyanin-conjugated MHC class I tetramers of H-2Db complexed with LCMV gp33–41 or np396–404 (both gifts of Dr. John Wherry, Wistar Institute). Splenocytes were re-stimulated *ex-vivo* with a pooled LCMV peptide at 2µg/mL (gift of Dr. John Wherry), which is a mixture of np396–404, np205–212, np166–175, np235–243, gp33–41, gp276–286, gp118–125, gp92–101, and gp70–77, plus golgi stop (BD intracellular stain kit) for 5 hours and the percentage of IFN-γ positive CD8 T cells was analyzed through flow cytometry.
